# Synthesis and Antimicrobial Activity of Some New Pyrazole, Fused Pyrazolo[3,4-d]-pyrimidine and Pyrazolo[4,3-e][1,2,4]-triazolo[1,5-c]pyrimidine Derivatives

**DOI:** 10.3390/molecules13071501

**Published:** 2008-07-29

**Authors:** Nada M. Abunada, Hamdi M. Hassaneen, Nadia G. Kandile, Omar A. Miqdad

**Affiliations:** 1 Department of Chemistry, Faculty of Applied Sciences, Al-Aqsa University, 76888 Gaza, Palestine; E-mail: miqdadomar@hotmail.com (O. A. Miqdad); 2 Department of Chemistry, Faculty of Science, Cairo University, Egypt; E-mail: hamdi_251@yahoo.com (H. M. Hassaneen); 3 Department of Chemistry, University College for Women, Ain Shams University, Heliopolis, Cairo, Egypt; E-mail: nadiaghk@yahoo.com (N. G. Kandile)

**Keywords:** Hydrazonyl bromides, pyrazolo[3,4-*d*]pyrimidine, pyrazolo[4,3-*e*][1,2,4]-triazolo[1,5-*c*]pyrimidine, antimicrobial activity

## Abstract

Hydrazonyl bromides **2a**,**b** reacted with active methylene compounds (dibenzoylmethane, acetylacetone, ethyl acetoacetate, phenacyl cyanide, acetoacetanilide, ethyl cyanoacetate, cyanoacetamide and malononitrile) to afford the corresponding 1,3,4,5-tetrasubstituted pyrazole derivatives **5-12a**,**b**. Reaction of **12a**,**b** with formamide, formic acid and triethyl orthoformate give the pyrazolo[3,4-*d*]pyrimidine, pyrazolo[3,4-*d*]pyrimidin-4(3H)one and 5-ethoxymethylene-aminopyrazole-4-carbo-nitrile derivatives **13-15a**,**b**, respectively. Compounds **15a**,**b** reacted with benzhydrazide and hydrazine hydrate to afford pyrazolo[4,3-*e*][1,2,4]triazolo[1,5-*c*]pyrimidine and [4-iminopyrazolo-[3,4-*d*]pyrimidin-5-yl]amine derivatives **16a**,**b** and **17a**,**b**. Reactions of compounds **17a**,**b** with triethyl orthoformate and carbon disulfide give the corresponding pyrazolo[4,3-*e*]-[1,2,4]triazolo[1,5-*c*]pyrimidine derivatives **18a**,**b** and **19a**,**b**, respectively.

## Introduction

Pyrazole and fused heterocyclic pyrazole derivatives constute an interesting class of heterocycles due to their synthetic versatility and effective biological activities [1,2,3]. Pyrazolo[3,4-*d*]pyrimidine derivatives have been found to possess antitumor and antileukemia activity [4,5,6,7], pyrazolo[4,3-e]-1,2,4-triazolo[1,5-c]pyrimidine derivatives have been found to be highly potent and selective human A3 [8,9,10,11], A2A [10] and A 2B [11] adenosine receptor antagonists. On the other hand, hydrazonyl halides have been shown to be biologically active depending on the nature of the groups on the carbon and nitrogen atoms. For example, *C*-acetyl and *C*-ethoxyhydrazonyl halides were reported to be active against red spider mites on beans and apple trees [12], whereas their *C*-aryl and *C*-aroyl analogues exhibit antiviral and antimicrobial [13] properties and *C*-alkylmethanohydrazonyl halides possess miticidal, insecticidal and herbicidal properties [14]. Moreover, many selectively fluoro-substituted organic compounds show peculiar pharmacological and agrOCHemical properties [15,16,17,18,19,20]. In continuation of our work concerning the preparation of hydrazonyl halides [21,22,23] and their reactions in the synthesis of heterocyclic compounds [24,25,26,27], we decided here, on one hand, to synthesize *C*-(4-fluorophenyl)- [28], and *C*-(2,4-dichlorophenyl)-*N*-(4-nitrophenyl)methanohydrazonyl bromides [29], which have received very little attention [29,30], surmising that both might have biological activity, and, on the other hand, to synthesize some new heterocyclic derivatives with anticipated biological activity.

## Results and Discussion

**Scheme 1:**
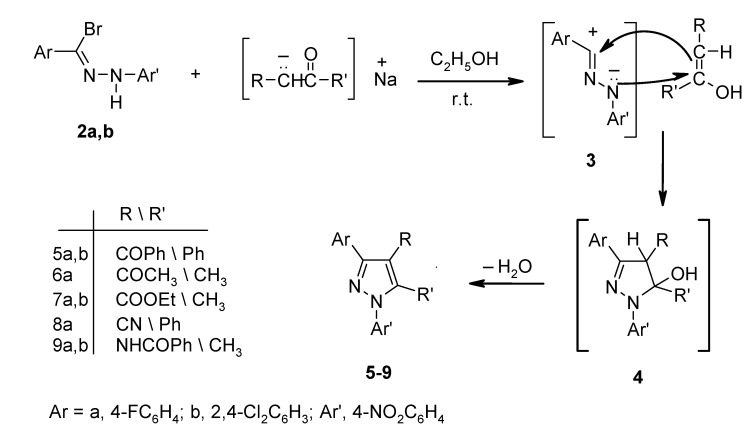


Reaction of hydrazonyl bromides **2a**,**b** with dibenzoylmethane in sodium ethoxide solution at room temperature gave, as the sole separable product, 3-aryl-4-benzoyl-1-(4-nitrophenyl)-5-phenylpyrazoles **5a**,**b** (Scheme 1).

The assignments of the structures of products **5a**,**b** were based on their correct elemental analyses and spectroscopic data. Their ^1^H-NMR spectra revealed only the expected aromatic proton multiplet signals at δ 7.1-8.4 ppm. The IR spectra of each product showed two characteristic absorption bands at 1644-1650 cm^-1^ and 1593-1594 cm^-1^, assignable to a conjugated benzoyl carbonyl and C=N groups, respectively.

Compound **5a** was also obtained when the nitrilimine **3a** [generated *in situ* from the reaction of triethylamine with hydrazonyl bromide **2a**] was reacted with dibenzoylmethane in tetrahydrofuran. This finding supports the mechanism suggested earlier for a similar reaction [29], whereby the carbanion – acting as a base – reacts with the hydrazonyl bromide to form a nitrilimine dipole **3**, which adds to the enol tautomers of the active methylene compounds used to form **4**, which then loses one molecule of water to give the pyrazole product.

**Scheme 2:**
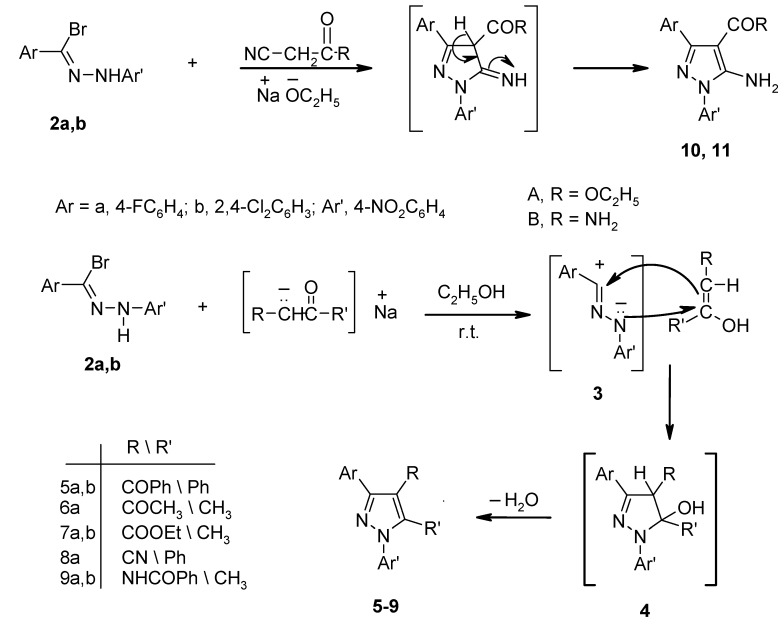


Acetylacetone, ethyl acetoacetate, phenacyl cyanide, acetoacetanilide reacted with **2a**,**b** under similar reaction conditions and gave the corresponding pyrazole derivatives **6-9**, respectively (Scheme 1). Similarly, **2a**,**b** reacted with ethyl cyanoacetate and cyanoacetamide to give ethyl 5-amino-3-aryl-1-(4-nitrophenyl)pyrazole-4-carboxylates **10a**,**b** and 5-amino-3-aryl-1-(4-nitrophenyl)-pyrazole-4-carboxamide derivatives **11a**,**b**, respectively (Scheme 2). The structures were confirmed by the correct elemental analyses (Table 2) and spectroscopic data (Table 3).

**Scheme 3:**
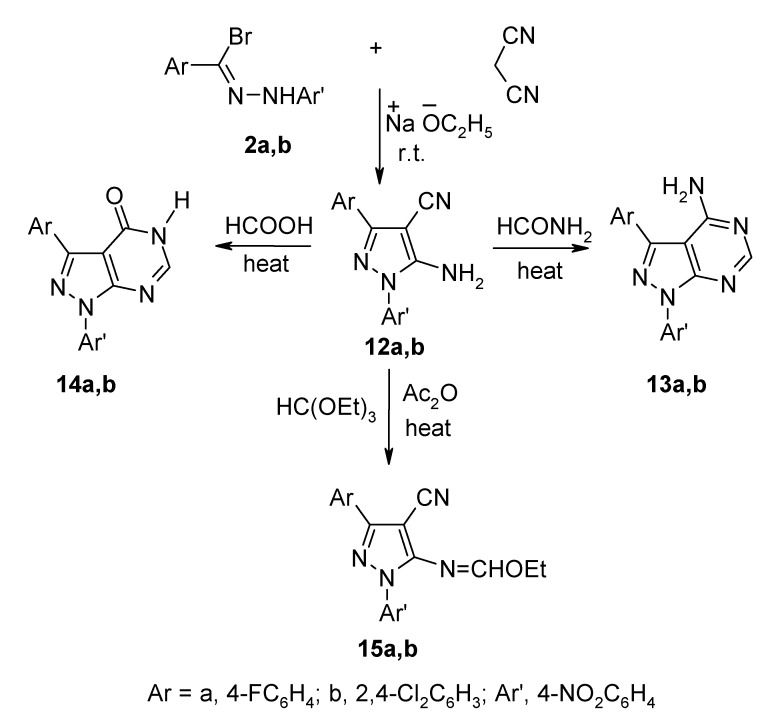


In addition, treatment of **2a**,**b** with malononitrile in the presence of sodium ethoxide afforded the 5-aminopyrazole-4-carbonitrile derivatives **12a**,**b** (Scheme 3). The structural assignments of these products were based on their elemental analyses and spectral data. Their IR spectra showed absorption bands near υ 3420-3300 cm^-1^, assignable to the NH_2_ stretching vibrations and a characteristic band near υ 2210 cm^‑1^, corresponding to a C≡N linkage. Their ^1^H-NMR spectra showed a characteristic signal near δ 6.80 ppm, assignable to the NH_2_ protons. The proposed structures of **12a**,**b** were also supported by their chemical reactivity. Thus, since they contain a β–enaminonitrile moiety, which is well known to be highly reactive, they were used as intermediates for the synthesis of new pyrazolo[3,4-d]pyrimidine derivatives. Refluxing compounds **12a,b** with excess formamide and formic acid gave the corresponding 4-amino-3-aryl-1-(4-nitrophenyl)pyrazolo[3,4-d]pyrimidine derivatives **13a,b** and 3-aryl-1-(4-nitrophenyl)pyrazolo[3,4-d]pyrimidin-4-one derivatives **14a,b**, respectively (Scheme 3). The IR spectra of **13** and **14** displayed no cyano group absorptions.

**Scheme 4:**
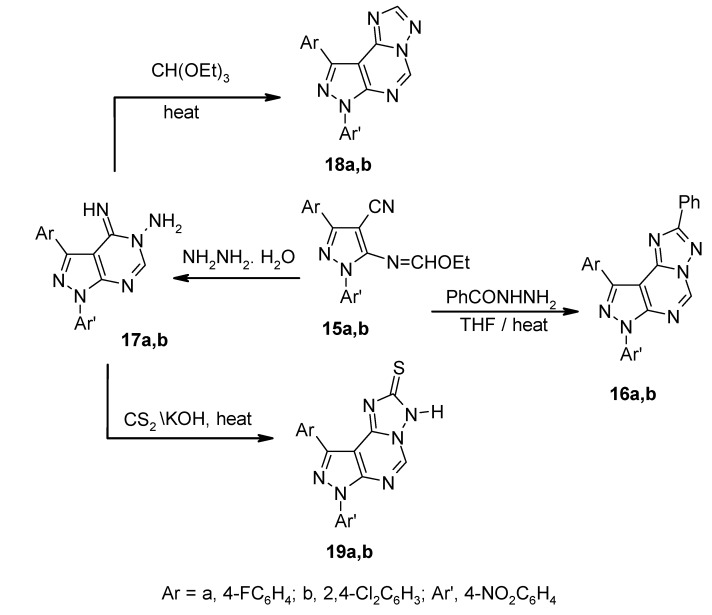


The condensations of compounds **12a**,**b** with triethyl orthoformate at reflux afforded the corresponding 5-ethoxymethyleneaminopyrazole-4-carbonitrile derivatives **15a**,**b**, respectively (Scheme 3). The confirmation of these structures was based on their analytical (Table 2) and spectroscopic data (Table 3). The structures of **15a**,**b** were further confirmed by their reactions with benzhydrazide and hydrazine hydrate. Thus, treatment with benzhydrazide in tetrahydrofuran at reflux temperature afforded **16a**,**b** (Scheme 4). The IR spectra of the products confirmed the disappearance of the nitrile absorption bands. The ^1^H-NMR spectra showed in each case a signal at δ 8.5 ppm, corresponding to the pyrimidine proton. Furthermore, when compounds **15a**,**b** were reacted with hydrazine hydrate in tetrahydrofuran at ambient temperature they produced the corresponding [3-aryl-4-imino-1-(4-nitrophenyl)-1,4-dihydro-5H-pyrazolo[3,4-*d*]pyrimidin-5-yl]amine derivatives **17a**,**b** (Scheme 4). The structures proposed for these products were established from their correct elemental analyses (Table 2) and spectroscopic data (Table 3). Their IR spectra revealed the absence of nitrile absorption frequencies. The ^1^H-NMR spectra for **17a**,**b** showed signals at δ 8.4 ppm, corresponding to the pyrimidine proton, and 5.0 ppm, assignable to the NH_2_ protons. The ^13^C-NMR spectrum of **17b** was also compatible with the proposed structure.

Further confirmation of the structures of **17a**,**b** was achieved from their reactions with triethyl orthoformate and carbon disulfide. Thus, refluxing compounds **17a**,**b** in an excess of triethyl orthoformate gave products **18a**,**b**, which were identified on the basis of correct elemental analyses and spectroscopic data as 9-aryl-7-(4-nitrophenyl)pyrazolo[4,3-*e*][1,2,4]-triazolo[1,5-*c*]pyrimidine derivatives (Scheme 4). The IR spectra of **18a**,**b** displayed no absorption bands for the NH and NH_2 _groups, which were observable in compounds **17a**,**b**. The ^1^H-NMR spectra showed signals near δ 9.7-9.8 and 9.4-9.5 ppm, assignable to the pyrimidine and the triazole proton, respectively. In addition, refluxing compounds **17a**,**b** with carbon disulfide and potassium hydroxide in ethanol gave **19a**,**b**, which were identified as 9-aryl-7-(4-nitrophenyl)pyrazolo[4,3-*e*][1,2,4]triazolo[1,5-*c*]pyrimidine-2-thione derivatives (Scheme 4). The structures of the compounds produced were established by spectroscopic data. The IR spectra revealed absorption bands corresponding to the NH and C=S functions near 3421.1 or 3422.4 and 1240 cm^-1^, respectively. The ^1^H-NMR spectra exhibited two singlets at δ 9.2 and 9.8 ppm, representing the protons of the pyrimidine and NH. Also, the mass spectra of all prepared compounds were compatible with the proposed structures (Table 3).

### Antimicrobial Activity

Some of the prepared compounds (namely **2a**,**b**, **6a**, **9b**, **17b** and **18b**) were screened for antibacterial activity (in nutrient agar broth) and antifungal activity (in Dox's medium and Saboured's agar) by the agar diffusion method [31,32] at a concentration 20 mg/mL using DMSO as solvent and blank. The compounds were tested for their activities against Gram +ve bacteria (*Staphylococcus aureus*) and Gram –ve bacteria (*Escherichia coli*), in addition to the pathogenic fungi *Aspergillus flavus* and *Candida albicans*. The antimicrobial screening results were measured by the average diameter of the inhibition zones, expressed in mm, and are presented in Table 1. As shown in the results, all the tested compounds displayed significant activities against *E. coli*, *S. aureus* and *C. albicans*, while only compound **2a** was very active against *A. flavus* and showed almost the same activity when compared with the usually used antifungal agents at the same concentration. Also, it was observed that the hydrazonoyl bromide **2a** has shown the highest activity toward all the tested organisms among all the tested compounds.

**Table 1:** Antimicrobial activities of the tested compounds

Sample	Inhibition zone diameter (mm / mg sample)			
	*E. coli* (G^-^)	*S. aureus* (G^+^)	*A. flavus* (Fungus)	*C. albicans* (Fungus)
Control (DMSO)	0.0	0.0	0.0	0.0
**2a**	15	16	13	14
**2b**	12	14	0.0	12
**6a**	13	13	0.0	12
**9b**	12	12	0.0	10
**17b**	13	13	0.0	12
**18b**	10	10	0.0	9
Tetracycline	32	34	--	--
Flucoral	--	--	14	16
Amphotricine	--	--	16	20

## Conclusions

In this report, the synthesis of new pyrazole derivatives **5-12** by the reaction of some active methylene compounds with hydrazonoyl bromides **2a,b** is reported. Also, the reaction of **12** with formamide, formic acid and triethyl orthoformate afforded the corresponding pyrazolo[3,4-d]pyrimidines **13**, **14** and 5-ethoxymethyleneaminopyrazole-4-carbonitriles **15**, respectively. Compound **15** reacted with benzhydrazide and hydrazine hydrate to give the corresponding pyrazolo[4,3-e][1,2,4]triazolo[1,5-c]pyrimidines **16** and 5H-pyrazolo[3,4-d]pyrimidin-5-yl]amines **17**. Also, **17** reacted with triethyl orthoformate and carbon disulfide and gave the corresponding pyrazolo[4,3-e][1,2,4]triazolo[1,5-c]pyrimidines **18** and **19**. The antibacterial and antifungal activity screening data of some of the prepared compounds showed promising antibacterial and antifungal activity. Compound **2a** is the most effective one and it also exhibited high activity against the fungus *A. flavus*.

## Experimental

### General

All melting points were measured on an Electrothermal melting point apparatus and are uncorrected. The infrared spectra were recorded in potassium bromide pellets on a Pye Unicam SP 3-300 or Shimadzu FT-IR 8101 PC infrared spectrophotometer. The ^1^H-NMR (200 MHz) and ^13^C-NMR (50 MHz) spectra were recorded in (DMSO-d_6_) on a GEMINI-200 spectrometer using TMS as the internal reference. Results are expressed in ppm and coupling constants (*J*) in Hertz. Mass spectra were measured on a GCMS-QP 1000 EX spectrometer at 70 eV. Elemental analyses were carried out at the Microanalytical Center of Cairo University, Giza, Egypt. Yields, analytical and spectroscopic data for the products are given in Table 2 and Table 3. The starting materials *C-(4-fluorophenyl)-N-(4-nitrophenyl)methanohydrazonyl bromide* [**2a** [28], yellow solid, mp. 224-6 °C (from tetrahydrofuran), lit. [28] mp. 202 °C] and *C-(2,4-dichlorophenyl)-N-(4-nitrophenyl)methanohydrazonyl bromide* [**2b**, [29], yellow solid, mp. 228-30 °C (from dioxane), lit. [29] mp. 212 °C] were prepared according to the indicated literature methods.

*Reaction of hydrazonyl bromides with active methylene compounds: General method for the synthesis of 4,5-disubstituted-3-aryl-1-(4-nitrophenyl)pyrazoles* **5-12**

The appropriate active methylene compound (dibenzoylmethane, acetylacetone, ethyl acetoacetate, phenacyl cyanide, acetoacetanilide, ethyl cyanoacetate, cyanoacetamide, malononitrile, 0.005 mol) was added with stirring to an ethanolic sodium ethoxide solution [20 mL, prepared from sodium metal (0.11 g, 0.005 mol) and absolute ethanol]. To the resulting solution the appropriate hydrazonyl bromide **2a**,**b** (0.005 mol) was added at room temperature. The mixture was stirred for 24 h, during which the bromide dissolved and the crude pyrazole precipitated. The latter was collected, washed with water, dried and crystallized from the indicated solvent.

*4-Benzoyl-3-(4-fluorophenyl)-1-(4-nitrophenyl)-5-phenylpyrazole* (**5a**). Off-white solid; mp. 185-8 °C (from acetic acid).

*4-Benzoyl-3-(2,4-dichlorophenyl)-1-(4-nitrophenyl)-5-phenylpyrazole* (**5b**). Pale yellow solid; mp. 159-61 °C (from ethanol); lit. mp. 150 °C [29].

*4-Acetyl-3-(4-fluorophenyl)-5-methyl-1-(4-nitrophenyl)pyrazole* (**6a**). Pale yellow crystals; mp. 149-51 °C (from acetic acid).

*Ethyl 3-(4-fluorophenyl)-5-methyl-1-(4-nitrophenyl)pyrazole*-4-carboxylate (**7a**). Off-white solid; mp. 162-4 °C (from ethanol).

*Ethyl 3-(2,4-dichlorophenyl)-5-methyl-1-(4-nitrophenyl)pyrazole*-4-carboxylate (**7b**). Pale yellow solid; mp. 114-6 °C (from ethanol); lit. mp. 121 °C [29].

*3-(4-Fluorophenyl)-1-(4-nitrophenyl)-5-phenylpyrazole*-4-carbonitrile (**8a**). Yellow crystals; mp. 188-90 °C (from dioxane-ethanol).

*3-(4-Fluorophenyl)-5-methyl-1-(4-nitrophenyl)-4-phenylaminocarbonylpyrazole* (**9a**). Yellow crystals; mp. 223-5 °C (from dioxane-ethanol).

*3-(2,4-Dichlorophenyl)-5-methyl-1-(4-nitrophenyl)-4-phenylaminocarbonylpyrazole* (**9b**). Pale brown solid; mp. 208-10 °C (from acetic acid).

*Ethyl 5-amino-3-(4-fluorophenyl)-1-(4-nitrophenyl)pyrazole-4-carboxylate* (**10a**). Pale brown solid; mp. 225-7 °C (from acetic acid); ^13^C-NMR: *δ* 164.2 (d, *J* = 253.4, C-F), 163.07 (C=O, ester), 151.50 (C=N), 151.41 (4-C pyrazole), 145.03 (C-*p*-NO_2_), 142.57 (C-NO_2_), 130.96 (d, *J* = 8.5, C-*m*-F), 128.74 (d, *J* = 3.4, C-*p*-F), 124.49, 123.24 (C-*o*, C-*m*-NO_2_), 114.10 (d, *J* = 21.6, C-*o*-F), 92.62 (C-5 pyrazole), 58.86 (OCH_2_CH_3_), 13.50 (OCH_2_CH_3_).

*Ethyl 5-amino-3-(2,4-dichlorophenyl)-1-(4-nitrophenyl)pyrazole-4-carboxylate* (**10b**). Yellow crystals solid; mp. 229-30 °C (from dioxane-ethanol).

*5-Amino-3-(4-fluorophenyl)-1-(4-nitrophenyl)pyrazole-4-carboxamide* (**11a**). Brownish yellow solid; mp.303-5 °C (from acetic acid).

*5-Amino-3-(2,4-dichlorophenyl)-1-(4-nitrophenyl)pyrazole-4-carboxamide* (**11b**). Yellow solid; mp. 261-3 °C (from dioxane-ethanol).

*5-Amino-3-(4-fluorophenyl)-1-(4-nitrophenyl)pyrazole-4-carbonitrile* (**12a**). Yellow solid; mp. 273-5 °C (from dioxane-ethanol).

*5-Amino-3-(2,4-dichlorophenyl)-1-(4-nitrophenyl)pyrazole-4-carbonitrile* (**12b**). Yellow solid; mp. 222-4 °C (from dioxane-ethanol).

*Alternate preparation of 4-benzoyl-3-(4-fluorophenyl)-1-(4-nitrophenyl)-5-phenyl-pyrazole* (**5a**).

A solution of triethylamine (0.7 ml, 0.005 mmol) in THF (5 mL) was added to a solution of hydrazonyl bromide **2a** (1.68 g, 0.005 mmol) and dibenzoylmethane (0.005 mmol) in THF (40 mL) and the resulting mixture was stirred at room temperature for 4 h. The solvent was then evaporated and the residue was triturated with ethanol whereupon it solidified. The solid was collected, washed with water (10 mL) and crystallized from acetic acid to give the product, which was identical in all respects (mp., IR, MS, ^1^H-NMR and elemental analysis) to compound **5a** prepared by the general method. Compound 5a was obtained in 59 % yield by this method.

*Synthesis of [3-aryl-1-(4-nitrophenyl)-1H-pyrazolo[3,4-d]pyrimidin-4-yl]amines* **13a,b**

A mixture of 5-amino-3-aryl-1-(4-nitrophenyl)pyrazole-4-carbonitrile **12a,b** (0.005 mol) and formamide (10 mL) was refluxed for 2-3 h. The solution was cooled, then poured onto water. The solid that precipitated was collected and crystallized to give *[3-(4-fluorophenyl)-1-(4-nitrophenyl)-1H-pyrazolo[3,4-d]pyrimidin-4-yl]amine* [**13a**, off-white solid; mp. >340 °C (from dimethylformamide] or *[3-(2,4-dichlorophenyl)-1-(4-nitrophenyl)-1H-pyrazolo[3,4-d]pyrimidin-4-yl]amine* [**13b**, off-white solid; mp. 347-8 °C (from DMF)].

*Synthesis of 3-aryl-1-(4-nitrophenyl)-1,4-dihydro-5H-pyrazolo[3,4-d]pyrimidin-4-ones* **14a**,**b**

A mixture of 5-amino-3-aryl-4-1-(4-nitrophenyl)pyrazole-4-carbonitrile **12a**,**b** (0.005 mol) and formic acid (20 mL) was refluxed for 1 h. The solution was cooled, and then poured on water. The solid that precipitated was collected and crystallized from the indicated solvent to give *3-(4-fluorophenyl)-1-(4-nitrophenyl)-1,4-dihydro-5H-pyrazolo[3,4-d]pyrimidin-4-one* [**14a**, off-white solid; mp. >340 °C (from dimethylformamide] or *3-(2,4-dichlorophenyl)-1-(4-nitrophenyl)-1,4-dihydro-5H-pyrazolo[3,4-d]pyrimidin-4-one* [**14b**, white solid; mp. >345 °C (from DMF)].

*Synthesis of 3-aryl-5-ethoxymethyleneamino-1-(4-nitrophenyl)pyrazole-4-carbonitriles* **15a**,**b**

A mixture of 5-amino-3-aryl-1-(4-nitrophenyl)pyrazole-4-carbonitrile **12a**,**b** (0.005 mol), triethyl orthoformate (3 mL) and acetic anhydride (20 mL) was heated under reflux for 5 h. After cooling the precipitated solid was filtered off and recrystallized from the indicated solvent to give *5-ethoxy-methyleneamino-3-(4-fluorophenyl)-1-(4-nitrophenyl)pyrazole-4-carbonitrile* [**15a**, white solid; mp. 194-6 °C (from tetrahydrofuran)] or *5-ethoxymethyleneamino-3-(2,4-dichlorophenyl)-1-(4-nitro-phenyl)pyrazole-4-carbonitrile* [**15b**, white solid; mp. 226-7 °C (from THF)].

*Synthesis of 9-aryl-7-(4-nitrophenyl)-2-phenyl-7H-pyrazolo[4,3-e][1,2,4]triazolo[1,5-c]pyrimidines* **16a**,**b**

A mixture of 3-aryl-5-ethoxymethyleneamino-1-(4-nitrophenyl)pyrazole **15a**,**b** (0.01 mol) and benzhydrazide (0.01 mol, 1.36 g) in THF (30 mL) was refluxed for 12 h. The mixture was cooled and the solvent was removed under reduced pressure and the resulting precipitate was purified by crystallization from the indicated solvent to give *9-(4-fluorophenyl)-7-(4-nitrophenyl)-2-phenyl-7H-pyrazolo[4,3-e][1,2,4]triazolo[1,5-c]pyrimidine* [**16a**, yellow solid; mp. >340 °C (from dimethylsulfoxide)] or *9-(2,4-dichlorophenyl)-7-(4-nitrophenyl)-2-phenyl-7H-pyrazolo[4,3-e]-[1,2,4]triazolo[1,5-c]pyrimidine* [**16b**, yellow solid; mp. 334-6 °C (from dioxane)].

*Synthesis of [3-aryl-4-imino-1-(4-nitrophenyl)-1,4-dihydro-5H-pyrazolo[3,4-d]pyrimidin-5-yl]amines* **17a**,**b**

Hydrazine hydrate (8 mL) was added to a suspension of 5-ethoxymethyleneamino-3-aryl-1-(4-nitrophenyl)pyrazole-4-carbonitriles **15a**,**b** (0.01 mol) in tetrahydrofuran (40 mL). The reaction mixture was stirred at room temperature for 1 h. The precipitate which formed was filtered off, washed with water, dried in air and crystallized from the indicated solvent to give *[3-(4-Fluorophenyl)-4-imino-1-(4-nitrophenyl)-1,4-dihydro-5H-pyrazolo[3,4-d]pyrimidin-5-yl]amine* [**17a**, pale yellow solid; mp. 266-8 °C (from DMF)] or *[3-(2,4-dichlorophenyl)-4-imino-1-(4-nitrophenyl)-1,4-dihydro-5H-pyrazolo[3,4-d]pyrimidin-4-yl]amine* [**17b**, pale yellow solid; mp. 256-8 °C (from DMF); ^13^C-NMR: *δ* 156.45 (C=N pyrimidine), 154.36 (C-5 pyrazole), 144.46, 144.27, 143.55, 134.98, 134.63, 134.21, 132.92, 129.36 (8C, ArC's), 127.50, 125.02, 124.98, 120.53, 120.21 (5C, ArCH's)].

*Synthesis of 9-aryl-7-(4-nitrophenyl)-7H-pyrazolo[4,3-e][1,2,4]triazolo[1,5-c]pyrimidines* **18a**,**b**

A mixture of [3-aryl-4-imino-1-(4-nitrophenyl)pyrazole-5-yl]amine **17a**,**b** (0.005 mol) with (15 ml) of triethyl orthoformate was refluxed for 1 h. After cooling, the precipitated product was collected by filtration and crystallized from the indicated solvent to give *9-(4-fluorophenyl)-7-(4-nitrophenyl)-7H-pyrazolo[4,3-e][1,2,4]triazolo[1,5-c]pyrimidine* [**18a**, pale yellow solid; mp. 304-6 °C (from DMF-ethanol)] or *9-(2,4-dichlorophenyl)-7-(4-nitrophenyl)-7H-pyrazolo[4,3-e][1,2,4]-triazolo[1,5-c]pyrimidine* [**18b**, pale yellow solid; mp. 314-6 °C (from DMSO-ethanol)].

*Synthesis of 9-aryl-7-(4-nitrophenyl)-7H-pyrazolo[4,3-e][1,2,4]triazolo[1,5-c]pyrimidine-2(3H)-thiones* **19a**,**b**

A mixture of [3-aryl-4-imino-1-(4-nitrophenyl)pyrazol-5-yl]amine **17a**,**b** (0.01 mol) with carbon disulfide (0.76 g, 0.01 mol) and potassium hydroxide (0.56 g, 0.01 mol) in ethanol (15 mL) was heated under reflux for 10 h. After removal of ethanol, water was added and the resulting alkaline solution was acidified with acetic acid and the precipitate formed collected by filtration, washed with water, dried and crystallized from the indicated solvent to afford *9-(4-fluorophenyl)-7-(4-nitrophenyl)-7H-pyrazolo[4,3-e][1,2,4]triazolo[1,5-c]pyrimidine-2(3H)-thione* [**19a**, yellow solid; mp. >350 °C (from DMSO-ethanol)] or *9-(2,4-dichlorophenyl)-7-(4-nitrophenyl)-7H-pyrazolo[4,3-e][1,2,4]-triazolo[1,5-c]pyrimidine-2(3H)-thione* [**19b**, orange solid; mp. 306-8 °C (from DMF-ethanol)].

**Table 2:** Analytical data for the newly prepared compounds.

Product	Yield %	Mol. Form. Mol. Wt.	Analysis(%) Calcd./Found			
			C	H	N	Cl
**2a**	76	C_13_H_9_BrFN_3_O_2_ 338.13	46.17 46.25	2.68 2.66	12.42 12.07	
**2b**	93	C_13_H_8_BrCl_2_N_3_O_2_ 389.03	40.13 40.25	2.07 2.20	10.80 10.55	18.22 18.04
**5a**	66	C_28_H_1__8_FN_3_O_3_ 463.45	72.56 72.43	3.91 4.04	9.06 8.94	
**5b**	62	C_28_H_17_Cl_2_N_3_O_3_ 514.35	65.38 65.25	3.33 3.45	8.16 8.16	13.78 13.74
**6a**	71	C_18_H_14_FN_3_O_3_ 339.32	63.71 63.52	4.15 4.22	12.38 12.27	
**7a**	64	C_19_H_16_FN_3_O_4_ 369.34	61.78 62.16	4.36 4.41	11.37 11.19	
**7b**	53	C_19_H_15_Cl_2_N_3_O_4_ 420.24	54.30 54.21	3.59 3.65	9.99 9.87	16.87 16.64
**8a**	48	C_22_H_13_FN_4_O_2_ 384.36	68.74 68.46	3.40 3.46	14.57 14.46	
**9a**	58	C_23_H_17_FN_4_O_3_ 416.40	66.33 65.96	4.11 4.17	13.45 13.39	
**9b**	47	C_23_H_16_Cl_2_N_4_O_3_ 467.30	59.11 58.97	3.45 3.49	11.98 11.76	15.17 15.05
**10a**	52	C_18_H_15_FN_4_O_4_ 370.33	58.37 58.36	4.08 4.18	15.12 14.89	
**10b**	56	C_18_H_14_Cl_2_N_4_O_4_ 421.23	51.32 51.55	3.35 3.51	13.30 13.19	16.83 16.71
**11a**	53	C_16_H_12_FN_5_O_3_ 341.29	56.30 56.29	3.54 3.61	20.05 19.87	
**11b**	51	C_16_H_11_Cl_2_N_5_O_3_ 392.19	48.99 49.10	2.82 3.01	17.85 17.63	18.07 18.16
**12a**	64	C_16_H_10_FN_5_O_2_ 323.28	59.44 59.37	3.11 3.01	21.66 20.98	
**12b**	61	C_16_H_9_Cl_2_N_5_O_2_ 374.18	51.35 51.35	2.42 2.47	18.71 18.45	18.95 18.86
**13a**	93	C_17_H_11_N_6_O_2_ F350.30	58.28 58.26	3.16 3.15	23.99 24.01	
**13b**	85	C_17_H_10_Cl_2_N_6_O_2_ 401.20	50.89 51.17	2.51 2.55	20.94 20.81	17.67 17.74
**14a**	88	C_17_H_10_N_5_O_3_ F351.29	58.12 58.15	2.86 2.88	19.93 19.90	
**14b**	83	C_17_H_9_Cl_2_N_5_O_3_ 402.19	50.76 50.85	2.25 2.33	17.41 17.36	17.63 17.55
**15a**	87	C_19_H_14_N_5_O_3_ F379.34	60.15 60.18	3.72 3.71	18.46 18.44	
**15b**	82	C_19_H_13_N_5_O_3_Cl_2_ 430.24	53.03 53.02	3.04 3.06	16.27 16.27	16.48 16.47
**16a**	54	C_24_H_14_N_7_O_2_ F451.41	63.85 63.88	3.12 3.10	21.72 20.71	
**16b**	52	C_24_H_13_N_7_O_2_Cl_2_ 502.31	57.38 57.40	2.60 2.61	19.52 19.51	14.11 14.10
**17a**	67	C_17_H_12_N_7_O_2_ F365.32	55.88 55.90	3.31 3.03	26.83 26.80	
**17b**	63	C_17_H_11_N_7_O_2_Cl_2_ 416.22	48.93 48.91	2.89 2.91	23.50 23.54	16.99 17.01
**18a**	56	C_18_H_10_N_7_O_2_ F375.31	57.60 57.58	2.68 2.66	26.12 26.15	
**18b**	61	C_18_H_9_N_7_O_2_Cl_2_ 426.21	50.72 50.70	2.12 2.14	23.00 22.98	16.63 16.66
**19a**	52	C_18_H_10_N_7_O_2_ SF407.38	53.06 53.03	2.47 2.46	24.06 24.07	
**19b**	53	C_18_H_9_N_7_O_2_SCl_2_ 458.28	47.17 47.19	1.97 2.00	21.39 21.41	15.47 15.49

**Table 3:** Spectroscopic data of the newly prepared compounds.

Product	MS	IR (KBr)		^1^H-NMR (DMSO-d_6_)
**2a**	339 (M^+^+2, 98.1), 337 (M^+^, 100.0), 339 (88.3), 290 (9.3), 259 (10.2), 188 (17.6), 122 (48.7), 95 (64.8), 90 (43.3), 75 (51.7), 63 (54.1), 51 (21.3), 50 (18.3).	3258.2 (NH), 3087.7 (CH-aromatic), 1595.6 (C=N).		7.25-8.54 (m, 8H, ArH), 10.54 (s, 1H, NH).
**2b**	391 (M^+^+4, 20.2), 389 (M^+^+2, 47.3), 387 (M^+^, 30.5), 309 (28.3), 307 (38.8), 262 (43.9), 173 (54.0), 137 (39.9), 100 (29.4), 90 (45.7), 63 (100.0), 50 (34.4).	3254.5 (NH), 3089.2 (CH-aromatic), 1595.3 (C=N).		7.23-8.56 (m, 7H, ArH), 10.25 (s, 1H, NH).
**5a**	463 (M^+^, 97.0), 386 (94.0), 340 (45.7), 207 (26.8), 105 (40.4), 77 (100.0), 51 (39.6).	3115.6, 3080.1 (CH-aromatic), 1644.1 (CO benzoyl), 1593.8 (C=N).		7.14-8.32 (m, 18H, ArH).
**5b**	514 (M^+^+2)-1, 0.5), 480 (39.5), 478 (100.0), 432 (20.9), 105 (8.2), 77 (25.4), 51 (8.2).	3082.2 (CH-aromatic), 1650.4 (CO), 1594.7 (C=N).		7.19-8.42 (m, 17H, ArH).
**6a**	340 (M^+^+1, 40.4), 339 (M^+^, 41.1), 324 (100.0), 278 (43.7), 279 (40.5), 117 (13.0), 76 (27.1), 50 (13.7).	3084.4 (CH-aromatic), 1668.7 (CO acetyl), 1593.5 (C=N).		2.45 (s, 3H, CH_3_), 2.61 (COCH_3_), 7.21-8.26 (m, 8H, ArH).
**7a**	369 (M^+^, 100.0), 324 (82.6), 278 (35.2), 117 (12.4), 76 (26.0), 50 (13.9).	3117.1, 3090.8 (CH-aromatic), 1706.3 (CO ester), 1594.4 (C=N).		1.19 (t, 3H, *J* = 7.2, COOCH_2_CH_3_), 2.65 (s, 3H, CH_3_), 4.21 (q, 2H, *J* = 7.2, COOCH_2_CH_3_), 7.23-8.45 (m, 8H, ArH).
**7b**	384 (M-35) (100.0), 386 (37.2), 356 (80.4), 367 (85.8), 311 (39.6), 310 (38.8), 117 (18.6), 76 (48.6), 51 (11.0), 50 (18.8).	3088.7 (CH-aromatic), 1699.7 (CO ester), 1595.5 (C=N).		1.20 (t, 3H, *J* = 7.3, COOCH_2_CH_3_), 2.64 (s, 3H, CH_3_), 4.22 (q, 2H, *J* = 7.3, COOCH_2_CH_3_), 7.24-8.45 (m, 7H, ArH).
**8a**	384 (M^+^, 100.0), 337 (34.3), 76 (15.7), 50 (11.8).	3068.2 (CH-aromatic), 2225.3 (C≡N), 1596.7 (C=N).		7.23-8.45 (m, 13H, ArH).
**9a**	416 (M^+^, 13.8), 324 (100.0), 278 (31.9), 65 (18.4).	3239.7 (NH), 3061.9 (CH-aromatic), 1657.6 (CO amide), 1596.2 (C=N).		2.63 (s, 3H, CH_3_), 7.14-8.27 (m, 13H, ArH), 11.60 (s, 1H, NH).
**9b**	466 (M^+^, 4.3), 468 (M^+^+2, 2.9), 431 (40.2), 374 (100.0), 328 (47.4), 117 (16.9), 65 (70.3), 50 (8.9).	3418.1 (NH), 3080.8 (CH-aromatic), 1656.4 (CO amide), 1596.6 (C=N).		2.64 (s, 3H, CH_3_), 7.16-8.27 (m, 12H, ArH), 11.60 (s, 1H, NH).
**10a**	370 (M^+^, 59.2), 324 (100.0), 277 (10.5), 146 (23.1), 90 (11.9), 76 (12.3), 63 (8.6), 50 (8.7).	3458.5, 3327.9 (NH_2_), 3112.8 (CH-aromatic), 1675.4 (CO ester), 1613.5 (C=N).		1.16 (t, 3H, *J* = 7.3, COOCH_2_CH_3_), 4.17 (q, 2H, *J* = 7.3, COOCH_2_CH_3_), 6.84 (s, 2H, NH_2_), 7.21-8.43 (m, 8H, ArH).
**10b**	420 (M^+^, 18.0), 422 (M^+^+2, 9.7), 385 (100.0), 311 (14.4), 196 (20.0), 136 (10.1), 90 (22.8), 76 (38.5), 63 (23.0), 50 (20.7).	3203.5, 3326.4 (NH_2_), 3076.4 (CH-aromatic), 1680.6 (CO ester), 1619.2 (C=N).		1.18 (t, 3H, *J* = 7.2, COOCH_2_CH_3_), 4.18 (q, 2H, *J* = 7.2, COOCH_2_CH_3_), 6.84 (s, 2H, NH_2_), 7.22-8.43 (m, 7H, ArH).
**11a**	341 (M^+^, 62.3), 324 (100.0), 325 (77.9), 278 (13.1), 146 (37.6), 122 (12.0), 95 (17.5), 90 (27.0), 76 (30.6), 63 (26.7), 50 (24.4).	3495.3, 3371.8, 3277.3 (two NH_2_), 3119.9 (CH-aromatic), 1643.1 (CO amide), 1585.9 (C=N).		6.83 (s, br., 2H, NH_2_), 7.19-8.43 (m, 10H, ArHs, NH_2_).
**11b**	393 (M^+^+2, 7.2), 391 (M^+^, 11.5), 356 (100.0), 357 (84.8), 310 (18.2), 91 (14.6), 76 (19.4), 63 (21.7), 50 (21.8).	3496.1, 3376.4, 3284.4 (two NH_2_), 3087.3 (CH-aromatic), 1646.5 (CO amide), 1588.3 (C=N).		6.83 (s, br., 2H, NH_2_), 7.21-8.45 (m, 9H, ArH, NH_2_).
**12a**	324 (M^+^+1, 100.0), 323 (M^+^, 97.2), 278 (14.8), 177 (13.6), 156 (16.4), 75 (23.6), 76 (26.9), 63 (18.3), 50 (23.5).	3429.5, 3300.7 (NH_2_), 3079.7 (CH-aromatic), 2209.1 (C≡N), 1600.8 (C=N).		6.84 (s, 2H, NH_2_), 7.14-8.27 (m, 8H, ArH).
**12b**	373 (M^+^, 100.0), 375 (M^+^+2, 82.6), 327 (12.3), 292 (41.5), 173 (13.8), 129 (20.0), 76 (63.6), 63 (42.6), 50 (53.4).	3447.7, 3320.8 (NH_2_), 3031.3 (CH-aromatic), 2217.9 (C≡N), 1596.7 (C=N).		6.86 (s, 2H, NH_2_), 7.12-8.27 (m, 7H, ArH).
**13a**	350 (M^+^, 100.0), 304 (9.8), 303 (12.1), 276 (5.4), 156 (14.5), 122 (6.3), 63 (7.6), 50 (7.8).	3480.8, 3290.2 (NH_2_), 3069.3 (CH-aromatic), 1653.2 (C=N).		5.02 (s, br., 2H, NH_2_), 7.33-8.58 (m, 9H, ArH, pyrimidine-H).
**13b**	402 (M^+^+2, 72.6), 400 (M^+^, 100.0), 319 (35.4), 161 (44.2), 92 (26.5), 91 (31.9), 77 (46.9), 63 (49.6), 51 (46.9), 50 (54.0).	3470.2, 3318.7 (NH_2_), 3070.6 (CH-aromatic), 1657.4 (C=N).		6.20 (s, 2H, NH_2_), 7.14-8.27 (m, 7H, ArH), 9.11 (s, 1H, pyrimidine).
**14a**	351 (M^+^, 100.0), 248 (10.0), 184 (31.6), 157 (17.2), 145 (16.7), 133 (10.6), 90 (10.6), 76 (19.3), 75 (19.2), 63 (19.8), 50 (31.1).	3318.2 (NH), 3042.5 (CH-aromatic), 1686.1 (C=O), 1590.1 (C=N).		7.29-8.50 (m, 9H, ArH, pyrimidinone-H), 12.72 (s, br., 1H, NH pyrimidinone).
**14b**	403 (M^+^+2, 11.8), 401 (M^+^, 17.6), 366 (100.0), 368 (36.5), 320 (24.9), 90 (12.8), 76 (23.5), 63 (21.4), 50 (32.1).	3446.3 (NH), 3071.3 (CH-aromatic), 1690.1 (CO), 1589.2 (C=N).		7.15-8.27 (m, 7H, ArH), 8.75 (s, 1H, pyrimidinone), 11.16 (s, 1H, NH pyrimidinone).
**15a**	379 (M^+^, 100.0), 351 (15.6), 350 (19.3), 323 (55.0), 276 (16.9), 145 (12.6), 76 (15.3), 50 (11.5).		3098.0 (CH-aromatic), 2997.7, 2945.9 (CH-aliphatic), 2232.4 (C≡N), 1630.5 (C=N).	1.35 (t, 3H, *J* = 7.0, CH_3_), 4.42 (q, 2H, *J* = 7.0, OCH_2_), 7.38-8.42 (m, 8H, ArH), 8.69 (s, 1H, N=CH).
**15b**	431 (M^+^+2, 71.3), 429 (M^+^, 89.1), 368 (37.0), 366 (100.0), 320 (51.3), 292 (27.3), 195 (27.2), 156 (19.7), 90 (22.8), 76 (56.1), 63 (31.5), 50 (48.0).		3088.3, 3004.5 (CH-aromatic), 2950.3 (CH-aliphatic), 2221.9 (C≡N), 1629.9 (C=N).	1.36 (t, 3H, *J* = 7.0, CH_3_), 4.43 (q, 2H, *J* = 7.0, OCH_2_), 7.61-8.44 (m, 7H, ArH), 8.70 (s, 1H, N=CH).
**16a**	451 (M^+^, 100.0), 393 (6.5), 284 (11.0), 95 (6.3), 77 (8.2), 76 (8.5), 50 (6.4).		3063.6, 3010.0 (CH-aromatic), 1635.2 (C=N).	7.43-8.12 (m, 13H, ArH), 8.56 (s, 1H, pyrimidine).
**16b**	503 (M^+^+2, 58.1), 501 (M^+^, 100.0), 466 (73.1), 420 (28.5), 291 (10.4), 290 (10.1), 289 (12.8), 153 (11.3), 127 (10.0), 103 (18.9), 90 (12.4), 77 (42.2), 76 (28.3), 63 (13.1), 50 (17.9).		3086.8 (CH-aromatic), 1643.7 (C=N).	7.42-8.08 (m, 12H, ArH), 8.54 (s, 1H, pyrimidine).
**17a**	365 (M^+^, 100.0), 350 (8.0), 290 (10.2), 157 (6.0), 114 (6.7), 95 (8.6), 76 (9.4), 75 (12.0), 63 (8.6), 50 (11.3).		3430.6-3353.5 (NH, NH_2_), 3067.1 (CH-aromatic), 1582.3 (C=N).	5.01 (s, br., 2H, NH_2_), 7.16-8.56 (m, 8H, ArH, NH), 8.45 (s, 1H, pyrimidine).
**17b**	417 (M^+^+2, 52.0), 415 (M^+^, 91.3), 382 (70.9), 380 (100.0), 363 (56.1), 334 (30.6), 307 (28.6), 215 (30.1), 187 (37.8), 150 (38.3), 122 (36.7), 114 (34.3), 90 (32.1), 77 (36.7), 76 (62.8), 63 (62.2), 50 (75.0).		3326.2-3270.3 (NH, NH_2_), 3082.5 (CH-aromatic), 1597.1 (C=N).	5.02 (s, br., 2H, NH_2_), 7.19-8.59 (m, 8H, ArH, NH), 8.47 (s, 1H, pyrimidine).
**18a**	375 (M^+^, 100.0), 329 (14.2), 275 (12.9), 208 (36.4), 90 (13.1), 75 (30.2), 63 (15.9), 50 (28.0).		3087.1 (CH-aromatic), 1629.7 (C=N).	7.58-8.59 (m, 8H, ArH), 9.43, 9.78 (two s, 2H, triazole, pyrimidine).
**18b**	427 (M^+^+2, 36.7), 425 (M^+^, 55.5), 390 (36.6), 344 (30.0), 337 (19.9), 298 (21.2), 257 (41.4), 215 (44.2), 187 (100.0), 145 (27.8), 136 (23.1), 123 (21.2), 90 (50.6), 75 (30.1), 63 (44.9), 50 (27.9).		3036.1, 3095.8 (CH-aromatic), 1635.7 (C=N).	7.62-8.66 (m, 7H, ArH), 9.52, 9.85 (s, 2H, triazole, pyrimidine).
**19a**	407 (M^+^, 65.1), 325 (95.9), 257 (13.0), 146 (100.0), 136 (19.0), 121 (26.0), 106 (12.3), 95 (27.9), 90 (34.6), 75 (33.8), 63 (27.9), 50 (42.4).		3421.1 (NH), 3077.2 (CH-aromatic), 1632.1 (C=N), 1242.3 (C=S).	7.56-8.66 (m, 8H, ArH), 9.24 (s, 1H, pyrimidine), 9.89 (s, 1H, NH).
**19b**	427 (M^+^+2(-32), 65.9), 425 (M^+^(-32), 100.0), 390 (56.0), 344 (41.1), 208 (14.5), 145 (16.7), 76 (26.2), 75 (23.6), 63 (18.6), 50 (32.4).		3422.4 (NH), 3096.9 (CH-aromatic), 1649.5 (C=N), 1246.2 (C=S).	7.54-8.67 (m, 7H, ArH), 9.22 (s, 1H, pyrimidine), 9.88 (s, 1H, NH).
